# Development of a hexahistidine-3× FLAG-tandem affinity purification method for endogenous protein complexes in *Pichia pastoris*

**DOI:** 10.1007/s10969-014-9190-1

**Published:** 2014-11-15

**Authors:** Toshiaki Higo, Noriyuki Suka, Haruhiko Ehara, Masatoshi Wakamori, Shin Sato, Hideaki Maeda, Shun-ichi Sekine, Takashi Umehara, Shigeyuki Yokoyama

**Affiliations:** 1 Department of Supramolecular Biology, Graduate School of Nanobioscience, Yokohama City University, 1-7-29 Suehiro-cho, Tsurumi, Yokohama, Kanagawa 230-0045 Japan; 2 RIKEN Systems and Structural Biology Center, 1-7-22 Suehiro-cho, Tsurumi, Yokohama, Kanagawa 230-0045 Japan; 3 Division of Structural and Synthetic Biology, RIKEN Center for Life Science Technologies, 1-7-22 Suehiro-cho, Tsurumi-ku, Yokohama, 230-0045 Japan; 4 Department of Interdisciplinary Science and Engineering, School of Science and Engineering, Meisei University, 2-1-1 Hodokubo, Hino, Tokyo 191-8506 Japan; 5 Department of Biophysics and Biochemistry, Graduate School of Science, The University of Tokyo, Bunkyo-ku, Tokyo 113-0033 Japan; 6 RIKEN Structural Biology Laboratory, 1-7-22 Suehiro-cho, Tsurumi-ku, Yokohama, 230-0045 Japan

**Keywords:** Epitope tagging, RNA polymerase, Transcription, Yeast

## Abstract

We developed a method for efficient chromosome tagging in *Pichia pastoris*, using a useful tandem affinity purification (TAP) tag. The TAP tag, designated and used here as the THF tag, contains a thrombin protease cleavage site for removal of the TAP tag and a hexahistidine sequence (6× His) followed by three copies of the FLAG sequence (3× FLAG) for affinity purification. Using this method, THF-tagged RNA polymerases I, II, and III were successfully purified from *P. pastoris*. The method also enabled us to purify the tagged RNA polymerase II on a large scale, for its crystallization and preliminary X-ray crystallographic analysis. The method described here will be widely useful for the rapid and large-scale preparation of crystallization grade eukaryotic multi-subunit protein complexes.

## Introduction

Multi-subunit protein complexes function in a variety of biological processes, including the biosynthesis and metabolism of genomic DNA and proteins, for cellular homeostasis [1]. Structural analyses of protein complexes provide the keys toward understanding their biological functions and regulatory mechanisms. However, in many cases, it is difficult to solve the high-resolution structures of protein complexes, because the methods to prepare large quantities of multi-subunit protein complexes with high homogeneity are limited. Hence, the biochemical preparation of a homogeneous protein complex of interest is one of the most important steps in these studies.

The most straightforward method to prepare a multi-subunit protein complex of interest is to purify an endogenous protein complex from a crude cell lysate [2]. This method has facilitated the large-scale preparation of several intact, multi-subunit protein complexes with crystallization-grade quality. For example, the X-ray crystallographic structures of the RNA polymerase (RNAP) II core complex [3] and the 20S proteasome core complex [4], purified from the budding yeast *Saccharomyces cerevisiae,* were solved. However, the yield of a purified protein complex of interest is primarily limited by its cellular abundance, and thus this procedure generally requires laborious and empirically designed purification steps, and an extremely large amount of cells, especially for the purification of a low-abundance protein complex. The reconstitution of recombinantly expressed protein subunits is an alternative method to overcome this problem [5]. For example, the crystal structure of the transcriptional Mediator head module, consisting of 7 recombinantly expressed subunits, was solved at 4.3 Å resolution [6]. However, it is still quite difficult to reconstitute multi-subunit protein complexes, especially those containing more than ten subunits, presumably due to difficulties in achieving nearly equal expression and/or solubility of all of the subunits, and/or tracing the intact complex formation in vitro.

Another approach to obtain crystallization-grade multi-subunit protein complexes is the tandem affinity purification (TAP) method. The TAP method enabled the rapid isolation of a highly pure protein complex from a crude cell lysate [7, 8]. This method can be performed under mild conditions, to preserve the integrity of the complex. However, only a few crystal structures of eukaryotic protein complexes prepared by the TAP method have been solved [9–11]. This is probably due to the difficulty in preparing a sufficient amount of cells, even in the case of the most productive organism, *S. cerevisiae*, utilized so far in the TAP method. Thus, it is necessary to develop a new TAP methodology for another eukaryotic species that can be cultured more densely than *S. cerevisiae*.

The methylotrophic yeast *Pichia pastoris* has been used as an important expression host for the large-scale production of recombinant proteins, in both industrial and academic settings [12]. Among eukaryotic model organisms, *P. pastoris* can be grown to the highest cell density in simple and inexpensive medium for shaking-flask culture or fermentation. This enables the preparation of a sufficient amount of cells, without any special equipment. By basic fermentation techniques in a controlled environment, it is possible to achieve ultra-high cell densities of *P. pastoris* (*e.g.* >100 g/L dry cell weight; >400 g/L wet cell weight; and >500 OD_600_ U/mL), which are typically about one order of magnitude higher than those of *S. cerevisiae* (*e.g.* 10–30 g/L) [13, 14]. The completion of the genome sequencing of *P. pastoris* [15] has now enabled the adaptation of the TAP strategy to this yeast. The TAP-tagging vector for *P. pastoris*, based on the original TAP tag (yTAP), is composed of two IgG-binding domains of protein A, a tobacco etch virus (TEV) protease cleavage site and a calmodulin-binding peptide domain, and its application has so far been limited to the functional characterization of protein complexes involved in peroxisome biogenesis [16]. However, yTAP has several disadvantages for the large-scale preparation of an intact protein complex. First, the large tag size, approximately 21 kDa, poses an inherent risk of impairing the protein structure and/or function. Second, the yield may be decreased, because the yTAP method requires proteolytic elution in the first purification step.

In this study, we developed an efficient chromosome tagging method in *P. pastoris* using a useful TAP tag, containing a hexahistidine (6× His) and three copies of FLAG (3× FLAG), to establish a general methodology for the rapid purification of endogenous large protein complexes suitable for X-ray crystallography. We demonstrated the utility of this methodology by the purification of several multi-subunit protein complexes, RNAPs I, II, and III, from *P. pastoris* cells. Furthermore, we performed the crystallization and preliminary X-ray crystallographic analysis of the RNAP II complex, to demonstrate that the purity of the protein complex prepared by this methodology is suitable for crystallization.

## Materials and methods

### Strains and growth media

*P. pastoris* wild-type strain X33 (Invitrogen) was used as the parental strain in this study, and was grown in YPD (1 % yeast extract, 2 % peptone, and 2 % dextrose). The number of cells was calculated according to the formula, 1 optical density at 600 nm wavelength (OD_600_) = 5 × 10^7^ cells/mL.

### Construction of pNS046_THF, a C-terminal THF-tagging vector in *P. pastoris*

**Fig. 1:**
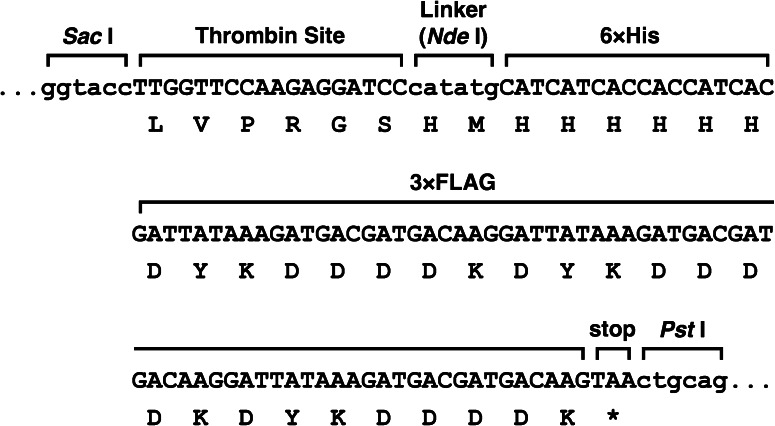
DNA and amino acid sequences of the THF-tag of the pNS046_THF plasmid

The p3FLAG-KanMX plasmid [17], containing three copies of the FLAG epitope sequence flanked by *Sac* I and *Pst* I digestion sites and the KanMX4 G418 resistance cassette, was used to construct pNS046_THF (Fig. 1). First, the additional *Sac* I site was disrupted by QuikChange site-directed mutagenesis (Stratagene), to produce pNS046. Subsequently, pNS046_THF was created by the insertion of the oligonucleotide (5′-*TTGGTTCCAAGAGGATCC*catatgCATCATCACCACCATCAC-3′) just upstream of the FLAG sequence of pNS046. This oligonucleotide sequence includes a thrombin protease cleavage site (italics), an *Nde* I site as a linker (lower case) and a hexahistidine tag sequence (underlined), respectively.

### Preparation of the DNA fragment for the transformation

**Fig. 2:**
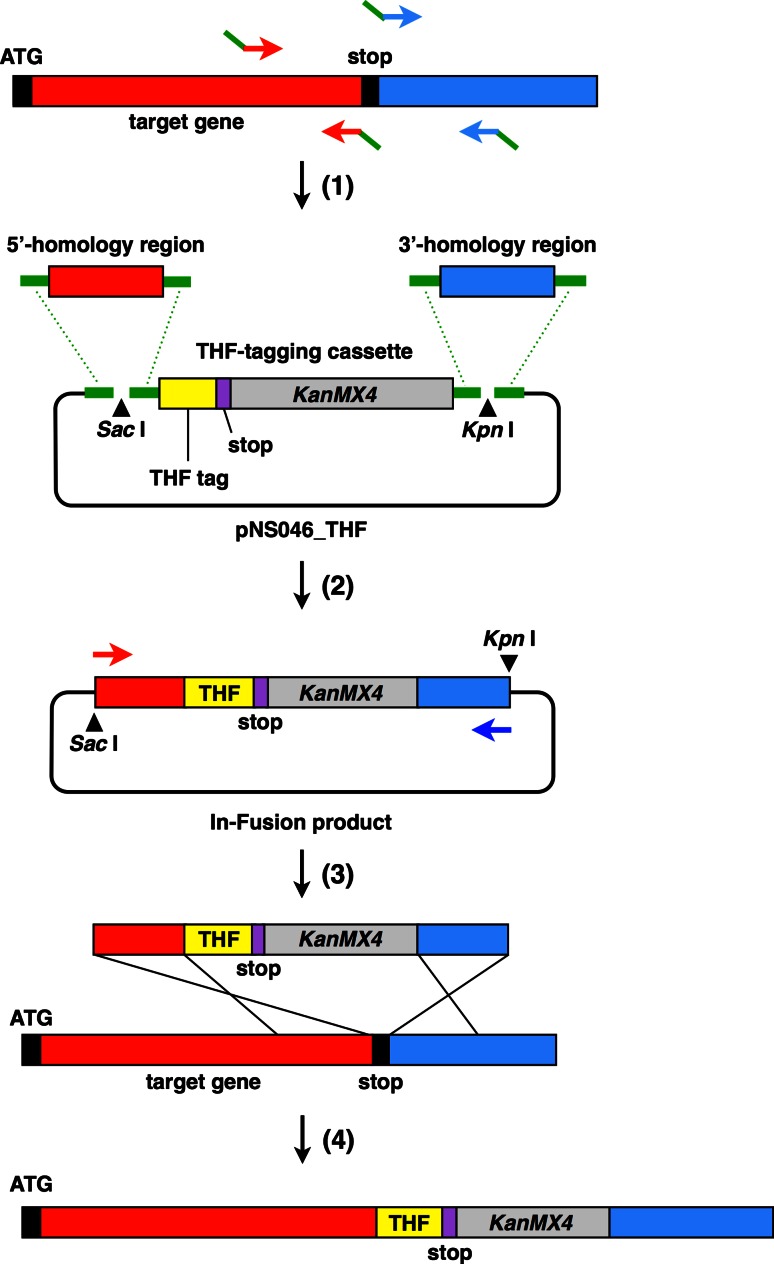
Schematic diagram of the integration of the THF tag DNA sequence into the genomic locus of the target protein at the C-terminus. **(** *1*) Preparation of homology region fragments. The approximately 800 bp PCR-amplified fragments upstream (5′-) or downstream (3′-) from the stop codon (excluding the stop codon) are indicated by *red* and *blue* *rectangles*, respectively. The 15 bp overlapping region at each end of the DNA fragment is shown by a *green bar*. (*2*) Assembly of the PCR fragments. 5′- (*red*) and 3′- (*blue*) homology regions and the THF-tagging cassette (*yellow* and *gray*) were joined to the *Sac* I- and *Kpn* I-digested pNS046_THF vector in a single In-Fusion reaction. (*3*) Homologous recombination. The DNA fragment for the transformation (*upper*), prepared by either digestion with *Sac* I and *Kpn* I or PCR amplification, was used for homologous recombination to introduce the THF tag to the C-terminus of the target protein. (*4*) Final THF-tagged chromosomal gene cassette in the *P. pastoris* genome

We used the In-Fusion HD Cloning Kit (TAKARA BIO) to construct the DNA fragment for the transformation of the THF-tagging cassette, which is sandwiched by approximately 800 bp tracts of 5′- and 3′-homology regions (Fig. 2). Genomic DNA from *P. pastoris* strain X33 was prepared using Dr. GenTLE for Yeast High Recovery (TAKARA BIO), according to the manufacturer’s protocol. For homologous recombination in *P. pastoris*, approximately 800 bp of the upstream (5′-) and downstream (3′-) regions from the stop codon were amplified from the genomic DNA by PCR, excluding the stop codon. For the subsequent In-Fusion reaction, the primers used in this PCR included a 15 bp overlap with the 5′-end of the segment-specific sequence. pNS046_THF was digested with *Sac* I and *Kpn* I and then two DNA fragments, corresponding to the THF-tagging module and the linear vector, were separately purified by agarose gel fractionation and extraction. The four DNA fragments (i.e. 5′- and 3′-homology region fragments, THF-tagging module, and the linear vector) were joined in a single In-Fusion reaction, utilizing the seamless in vitro assembly at the specific 15 bp overlap at their ends. The resultant construct was digested with *Sac* I and *Kpn* I, and the linearized insert DNA fragment for the transformation was purified. When the *Sac* I and *Kpn* I sites were present in both homology arms, the DNA fragment was amplified by PCR, using high-fidelity PrimeSTAR Max DNA Polymerase (TAKARA BIO).

### *P. pastoris* transformation using lithium chloride

A fresh single colony of X33 was inoculated in 5 mL of YPD and grown to saturation at 30 °C for 2 days. This pre-culture was diluted to an OD_600_ of 0.1–0.2 in 50 mL of fresh YPD and cultured until the OD_600_ reached 0.8–1.2. The cells were pelleted, washed with 1 mL of distilled water, and then washed with 1 mL of SORB [10 mM Tris–HCl buffer (pH 8.0), containing 1 mM EDTA, 1 M sorbitol, and 100 mM lithium chloride]. The pellet was suspended in 360 µL SORB, containing 40 µL sheared and denatured salmon sperm DNA (8 mg/mL in TE, Sigma) as a carrier. After vortex mixing, aliquots (50 µL) of these chemically competent cells were stored at −80 °C. For each transformation, 1.5 µg of the linearized DNA fragment, dissolved in 5 µL of distilled water, was added to 50 µL of thawed competent cells, and then mixed vigorously with 300 µL of freshly prepared PEG solution [10 mM Tris–HCl buffer (pH 7.5), containing 40 % PEG 4,000, 1 mM EDTA, and 100 mM lithium chloride]. After an incubation at 30 °C for 15 min with agitation, 35 µL of DMSO was added to the cell suspension, to a final concentration of 10 %. For the heat shock, the suspension was incubated at 42 °C for 15 min. After centrifugation, the supernatant was completely removed. The cell pellet was resuspended in fresh YPD medium and then spread onto YPD plates. After an incubation at 30 °C for 16 h, the plates were replica-plated on YPD plates containing 500 µg/mL G418 (Nacalai Tesque), and further incubated at 30 °C to select transformants.

### *P. pastoris* transformation by electroporation

The X33 pre-culture was diluted to an OD_600_ of 0.2 in fresh YPD and cultured until the OD_600_ reached 1.5–2.0. For each transformation, 8 × 10^8^ cells were washed twice with 1 mL of distilled water and were incubated in 8 mL of 10 mM Tris–HCl buffer (pH 7.5), containing 0.6 M sorbitol, 10 mM DTT, and 100 mM lithium acetate, for 30 min at room temperature. The cells were pelleted, washed three times with 1 mL of ice-cold 1 M sorbitol, and resuspended in ice-cold 1 M sorbitol at a final concentration of 10^8^ cells/mL, as electro-competent cells. An 80 µL aliquot of competent cell suspension was mixed vigorously with 10 ng of the DNA fragment for the transformation in 1 µL of distilled water, transferred to an ice-cold sterile electroporation cuvette (2.0 mm electrode gap; BIO-RAD), and incubated for 5 min on ice. Electroporation was performed using a Gene Pulser (BIO-RAD) at 1.5 kV, 25 µF, and 200 Ω. After application of the pulse, the cells were immediately diluted with 1 mL of ice-cold 1 M sorbitol, and were incubated for 30 min at 30 °C. The cell suspension was spread onto YPDS (YPD supplemented with 1 M sorbitol) plates. After an incubation at 30 °C for 16 h, the plates were replica-plated on YPDS plates containing 500 µg/mL G418 and further incubated at 30 °C. Transformed yeast colonies appeared in 2–3 days in the presence of 500 µg/mL G418. Colony-direct PCR, using SapphireAmp Fast PCR Master Mix (TAKARA BIO), was performed to identify the fusion of the THF-tag into the desired genome loci. Clones verified by colony-direct PCR were further analyzed by western blotting with an anti-FLAG M2 monoclonal antibody (Sigma), to detect the THF-tagged protein. Whole cell extracts were prepared by the post-alkaline method, as previously described [18].

### Purification of C-terminally THF-tagged proteins

The cells were grown for 2 days at 30 °C in YPD, which resulted in an OD_600_ approximately between 10 and 30. After harvest, the cells were washed once in distilled water and then resuspended in an equal volume (wet weight of cells/volume; g/mL) of lysis buffer [50 mM Tris–HCl buffer (pH 8.0), containing 150 mM NaCl, 1 mM EDTA, 10 mM magnesium sulfate, 50 mM ß-glycerophosphate, 0.5 % Triton X-100, and 1× complete mini EDTA-free protease inhibitor (Roche)]. The cell slurry was mixed with an equal volume of 0.5 mm diameter zirconia ceramic beads (Yasui Kikai), and whole cell extracts were prepared by bead beating with a Multi-beads shocker (Yasui Kikai) at 0 °C. Cellular debris was removed by centrifugation at 27,700×*g* for 30 min. The soluble fraction was collected and passed through a 0.45 µm filter membrane. Anti-FLAG M2 affinity gel (Sigma), equilibrated with lysis buffer, was added to the clarified extract, and the suspension was rocked overnight at 4 °C. The beads were washed with 15 volumes of lysis buffer and then with 10 volumes of 50 mM Tris–HCl buffer (pH 8.0). The bound proteins were eluted with an equal volume of 50 mM Tris–HCl buffer (pH 8.0), containing 200 µg/mL 3× FLAG peptide (Sigma). For further purification, an equal volume of Ni-equilibration buffer (300 mM NaCl, 20 % glycerol, and 0.2 % Tween-20) was added to the eluate from the anti-FLAG M2 affinity gel. This solution was then transferred to Ni Sepharose High Performance (GE Healthcare) beads, equilibrated with Ni-binding buffer [25 mM Tris–HCl buffer (pH 8.0), containing 150 mM NaCl, 10 % glycerol, 0.1 % Tween-20, and 1× complete mini EDTA-free protease inhibitor], and the suspension was rocked for 1 h at 4 °C. The beads were washed with 10 volumes of Ni-binding buffer. The bound protein complex was eluted with sequential step-elutions of 20, 500, and 1,000 mM imidazole in Ni-binding buffer. We also used the Ni-binding buffer containing 50 mM imidazole through the optimization of purification condition.

### Large-scale purification of RNA polymerase II

Approximately 400 g of cells were resuspended in an equal volume of lysis buffer [40 mM Tris–HCl buffer (pH 7.5), containing 100 mM potassium acetate, 1 mM EDTA, 5 mM DTT, 0.1 % Triton X-100, 0.5 mM PMSF, and 3× complete EDTA-free protease inhibitor] and then disrupted with an APV-2000 high-pressure homogenizer (SPX). The lysate was cleared by centrifugation, and the supernatant was loaded onto a Q Sepharose Fast Flow column (GE Healthcare) equilibrated with lysis buffer. After extensive washing of the column with wash buffer [20 mM Tris–HCl buffer (pH 7.5), containing 100 mM potassium acetate, 1 mM EDTA, and 0.1 % Triton X-100], the protein was eluted with buffer K1000 [20 mM Tris–HCl buffer (pH 7.5), containing 1,000 mM potassium acetate]. The eluted fractions were then loaded onto an anti-FLAG M2 affinity gel column. The column was washed with buffer K500 [20 mM Tris–HCl buffer (pH 7.5), containing 500 mM potassium acetate] and the fraction containing RNAP II was eluted with buffer K500 containing 100 μg/mL 3× FLAG peptide. The eluted RNAP II was further purified using a Ni Sepharose Fast Flow column (GE Healthcare). The column was washed with buffer K500 containing 10 mM imidazole, and the RNAP II-containing fractions were eluted with buffer K500 containing 90 mM imidazole. Finally, the protein was purified by anion-exchange column chromatography on a Resource Q column (GE Healthcare), eluted with a linear gradient from buffer A [20 mM Tris–HCl buffer (pH 7.5), containing 0.1 mM tris(2-carboxyethyl)phosphine (TCEP)] to buffer B [20 mM Tris–HCl buffer (pH 7.5), containing 1 M potassium acetate, and 0.1 mM TCEP]. The RNAP II-containing fractions were buffer-exchanged to RNAP II buffer [20 mM Tris–acetate buffer (pH 8.0), containing 150 mM potassium acetate, 4 mM magnesium acetate, 2 µM zinc acetate, and 4 mM TCEP], and then concentrated to 6 mg/mL with a Vivaspin 2 filter (10 kDa molecular mass cut-off; GE Healthcare).

### Crystallization of RNA polymerase II

Crystallization experiments were performed using the sitting-drop vapor diffusion method at 293 K. The initial screening was performed with the PGA Screen kit (Molecular Dimensions), and crystals were obtained under conditions containing 0.2 M sodium citrate and 8 % poly-γ-glutamic acid low molecular weight polymer (PGA-LM). A preliminary diffraction check was performed at the beam line BL32XU in SPring-8 (Harima, Japan).

## Results

### Design of the C-terminal THF-tagging sequence

In order to develop a methodology for the rapid and crystallization-quality preparation of a multi-subunit protein complex of interest in *P. pastoris*, we first constructed the novel THF-tagging vector, pNS046_THF, based on the p3FLAG-KanMX plasmid [17], which harbors three copies of the FLAG epitope sequence (DYKDDDDK) (3× FLAG) and a *KanMX4* G418 resistance cassette, by inserting a sequence containing the thrombin protease digestion site and the hexahistidine sequence (6× His) just upstream of the 3× FLAG sequence (Fig. 1). The size of the THF tag (4.7 kDa) is approximately one-fourth of that of yTAP. To purify both high- and low abundant proteins, or multi-subunit protein complexes, this combination of tagging system has been successfully applied to various model species [19–23]. We took advantage of this combinatorial tagging system for large-scale preparation of endogeneous multi-subunit protein complexes from *P. pastoris*.

### Homologous integration of the C-terminal THF-tagging sequence in *P. pastoris*

To examine the length of the homology sequence sufficient for homologous recombination in *P. pastoris*, we chose the gene encoding GDP dissociation inhibitor 1 (*GDI1*) [24] as a trial target, and constructed several C-terminal THF-tagging cassettes for *GDI1* that are sandwiched by different lengths of the 5′- and 3′-regions around the stop codon of *GDI1*. We then transformed *P. pastoris* X33 cells by the lithium method [25], using lithium chloride instead of lithium acetate, according to the *Pichia* Expression Kit (Invitrogen) user manual. The transformants with the THF-tagging cassette precisely inserted at the desired C-terminus of *GDI1* were initially selected by colony-direct PCR, and subsequently confirmed by western blotting of the whole cell extracts, using an anti-FLAG M2 monoclonal antibody. We found that the *GDI1* locus was refractory to the integration of the THF-tagging cassette when the flanking homologous sequences were shorter than ~100 bp. On the other hand, we obtained the desired transformants when THF-tagging cassettes with longer 5′- and 3′-homologous sequences (≥ ~100 bp) were used (Table 1). For efficient C-terminal epitope tagging in *P. pastoris*, we established a rapid procedure for the subcloning and preparation of a THF-tagging cassette, sandwiched by long tracts of PCR-generated homologous sequences, by a ligation-free cloning technique [26] (Fig. 2).

**Table 1:** Homologous integration of the THF-tagging sequence at *GDI1*

Length of homology (bp)		Correct insertion^a^
5′-region	3′-region	
1,923	700	+
790	564	+
608	467	+
378	368	+
264	173	+
102	130	+
102	82	−
40	40	−

^a^Correctly inserted clone was: (+): obtained; (−): not obtained

To establish a more efficient chromosome-tagging method in *P. pastoris*, we tested the electroporation method by transforming electrocompetent X33 cells, as previously described [27]. We compared the transformation efficiencies of the lithium method and the electroporation method, using the THF-tagging cassette sandwiched by approximately 800 bp 5′- and 3′-homologous sequences, in the genes encoding RNAP I-, II-, and III-specific subunits, *RPA135*, *RPB2*, and *RPO31*, respectively. In the case of *RPA135*, which encodes the second-largest subunit, RNAP I, the homologous recombinant, designated as NSY471, was successfully obtained by both methods. However, in the cases of *RPB2,* encoding the second-largest subunit of RNAP II, and *RPO31,* encoding the largest subunit of RNAP III, the homologous recombinants, designated as THY46 and THY48, respectively, were only obtained by the electroporation method. All four of the THF-tagged strains exhibited good growth phenotypes, and three of them (i.e. NSY471, THY46, and THY48) were subjected to high-density cell culture for the preparation of RNAPs from whole cell extracts.

### Tandem affinity purification of THF-tagged *P. pastoris* RNA polymerases

**Fig. 3:**
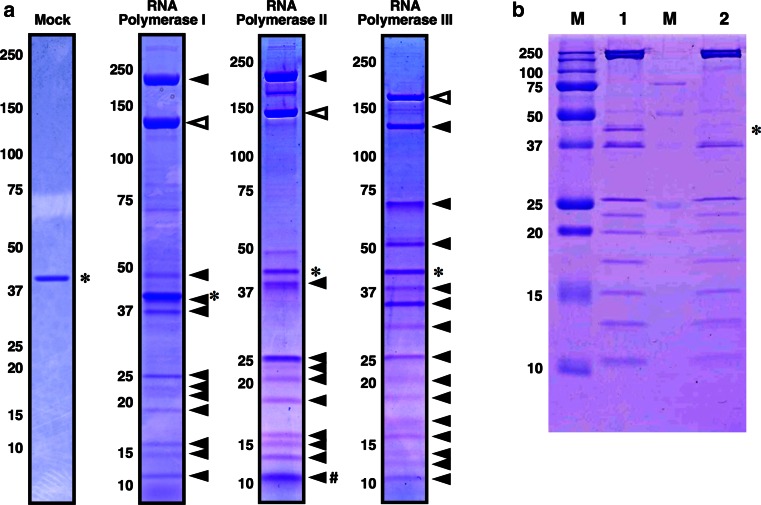
Purification of the RNA polymerase complexes from *P. pastoris.* (**a**) Purified RNAP complexes I, II and III. Proteins co-purified (*filled triangles*) with each THF-tagged RNAP subunit (*open triangles*) were separated on a 4–20 % sodium dodecyl sulfate (SDS) polyacrylamide gel and visualized by Coomassie Brilliant Blue (CBB) staining. In each complex, the putative RNAP subunits were judged by their predicted molecular weights. The non-specific band corresponding to Aro2p is marked with an *asterisk*. An untagged control strain is shown as “Mock”. The molecular weights (kDa) are shown on the *left*. In the RNAP II panel, two small subunits (i.e., Rpb10 and Rpb12) were not separated by the SDS-PAGE. Rpb10 (8.5 kDa) and Rpb12 (7.9 kDa) seemed to co-migrate at the position marked with *#*, as judged from their molecular weights. (**b**) Comparison of single and double affinity purification. Lane 1, eluate after purification with anti-FLAG M2 affinity gel; lane 2, eluate after purification with anti-FLAG M2 affinity gel and Ni Sepharose; and M, molecular weight markers (kDa). Electrophoresis was performed on a 15 % SDS polyacrylamide gel. Aro2p is indicated with an *asterisk*

To validate the His-FLAG TAP system for the purification of a *P. pastoris* protein complex, we tried to purify the endogenous *P. pastoris* RNAPs I, II, and III, using the strains NSY471, THY46, and THY48, respectively. Each strain was cultured in 100 mL of YPD medium, and approximately 2–4 g of cells were harvested. The soluble fraction from the whole cell extracts was loaded on an anti-FLAG M2 affinity gel column, and the THF-tagged protein and its associated factors were co-eluted with 3× FLAG peptide. The eluent was further affinity-purified with nickel-charged affinity resin. The proteins that co-purified with each THF-tagged RNAP subunit are shown in Fig. 3a. The subunit compositions of the purified *P. pastoris* RNAPs are identical to those of *S. cerevisiae* [28–30]. An approximate 40 kDa band was commonly detected (shown by asterisks in Fig. 3a) even in the purification of whole cell extracts from the non-tagged wild-type strain (Fig. 3a, mock), indicating that this protein bound non-specifically to the resins. The protein was identified by mass spectrometry as *P. pastoris* chorismate synthase Aro2p.

In the TAP purification, the Aro2p protein was a prominent contaminant in all of the tested cases (Fig. 3a). Therefore, we optimized the purification conditions using the RNAP II sample, to investigate whether Aro2p can be completely removed by the TAP purification. The Aro2p protein was not completely removed by the first step of the purification, using the anti-FLAG M2 affinity gel (Fig. 3b, lane 1). Using this RNAP II sample, we performed a second affinity purification with a Ni Sepharose column. Through optimization of the binding, washing and elution conditions, we were able to prepare an RNAP II sample that is nearly devoid of Aro2p (Fig. 3b, lane 2), by increasing the imidazole concentration to 50 mM during the washing steps.

### Crystallization of the THF-tagged *P. pastoris* RNA polymerase II

**Fig. 4:**
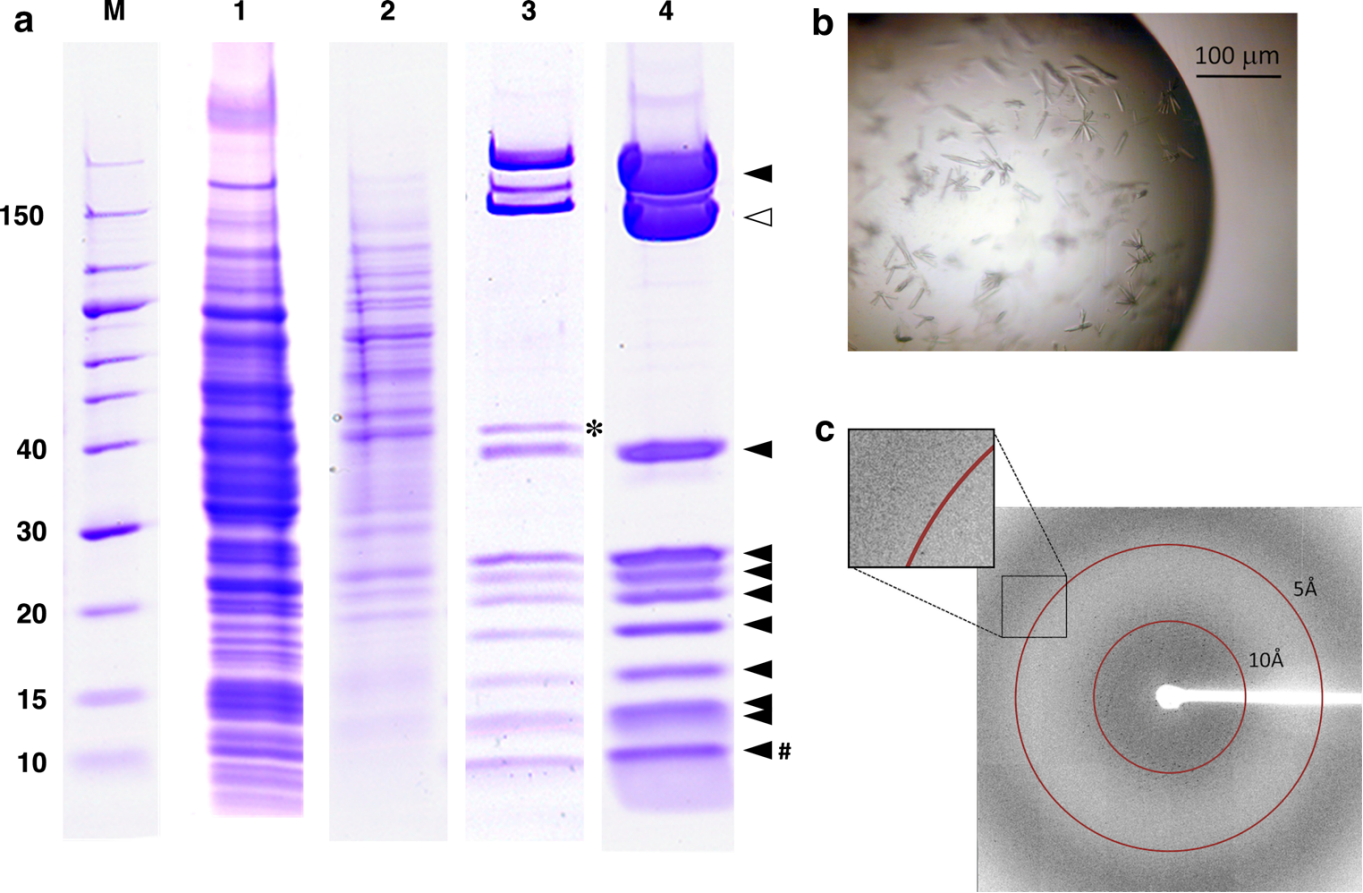
Large-scale purification and crystallization of the *P. pastoris* RNA polymerase II. (**a**) Large-scale purification of RNAP II. Protein fraction of each purification step was separated on a 10–20 % SDS polyacrylamide gel and visualized by CBB staining. M, molecular weight markers. The molecular weights (kDa) are shown on the *left*. Lane 1, total cell lysate; lane 2, elution from Q Sepharose; lane 3, elution from the TAP purification; and lane 4, elution from Resource Q. Aro2p is marked with an *asterisk* in lane 3. In lane 4, the THF-tagged Rpb2 subunit and its co-purified subunits are marked with an *open triangle* and *filled triangles*, respectively. Two small subunits, Rpb10 (8.5 kDa) and Rpb12 (7.9 kDa), seemed to co-migrate at the position marked with #. This figure is composed of multiple gel images. (**b**) Single crystals of *P. pastoris* RNAP II. (**c**) X-ray diffraction image of an RNAP II crystal

Finally, we performed the large-scale purification and crystallization of RNAP II, to demonstrate the advantages of this strategy for X-ray crystallography. Although we found that Aro2p could be almost completely removed by the TAP purification, through careful optimization of the purification conditions (Fig. 3b), we employed conventional anion-exchange column chromatography using a Resource Q column after the TAP purification, to completely remove the contaminating Aro2p in the large-scale purification (Fig. 4a). This combinatorial purification also enabled us to prepare the RNAP II sample devoid of Aro2p (Lane 4 in Fig. 4a). The purification scheme is shown in Table 2. Using this RNAP II sample and commercially available crystallization screening kits, we successfully obtained single crystals (Fig. 4b). Preliminary X-ray diffraction experiments using synchrotron radiation revealed that the obtained RNAP II crystals diffract up to 4.5 Å resolution (Fig. 4c). The crystals belong to the space group *P2*_*1*_, with unit cell dimensions of a = 155, b = 160, c = 254 Å, and β = 105°. Molecular replacement with the coordinates of *S. cerevisiae* RNAP II revealed that the asymmetric unit of the above crystal contains two RNAP II molecules comprising all 12 subunits (to be published elsewhere). These results demonstrate that the methodology described in this study leads to the crystallization-quality preparation of a eukaryotic multi-subunit protein complex.

**Table 2:** Purification of THF-tagged RNA polymerase II for crystallization

Purification step	Purity (%)^a^	Yield (mg)
Lysate (from 400 g wet cell weight)	<1	N.D.^b^
Q Sepharose	<1	N.D.
Anti-FLAG M2/Ni Sepharose tandem affinity	90	2.0
Resource Q	>95	1.5

^a^Assessed by SDS–polyacrylamide gel electrophoresis (PAGE) and CBB staining

^b^Not determined

## Discussion

The homogeneous preparation of an intact multi-subunit protein complex is one of the most fundamental requirements in biochemistry and protein science. Many huge protein complexes function in eukaryotic cells, such as DNA- and RNA polymerases, chromatin-associated complexes, spliceosomes, ribosomes, and proteasomes. The functional and single-particle structural analyses of these huge protein complexes have been performed for most of these complexes, but limited numbers of complexes have been subjected to high-resolution X-ray crystallographic analyses. The methodology developed in this study enabled the purification of a eukaryotic multi-subunit protein complex from *P. pastoris*, with crystallization quality.

Using this new method, we demonstrated that different multi-subunit protein complexes can be purified from TAP-tagged *P. pastoris* cultures. Regarding the target tagging subunit, we chose one of RNA polymerase-specific subunits, excluding five common subunits of Rpb5 (24.9 kDa), Rpb6 (17.7 kDa), Rpb8 (16.2 kDa), Rpb10 (8.5 kDa) and Rpb12 (7.9 kDa), and the two RNAP I/III-common subunits (AC40 and AC19). Among the remaining candidate RNAP subunits, we targeted the largest subunit (i.e., Rpo31 for RNAP III) or the second largest subunits (i.e., Rpa135 for RNAP I and Rpb2 for RNAP II) for the co-purification of the respective RNAP complexes. We chose these subunits because we expected that the TAP tag attached to one end of a larger subunit may have a higher chance of being exposed to the solvent, or a lower chance of disturbing the structural integrity of the multi-subunit complex.

In the large-scale purification for crystallization, we obtained approximately 1.5 mg of the highly-purified RNAP II complex from 400 g wet cell lysate, prepared from 4 l of *P. pastoris* culture (Fig. 4; Table 2). Such a large-scale culture of the yeast *P. pastoris*, in which a protein of interest is THF-tagged by the present methodology, may thus be generally practical for the crystal structure analyses of other protein complexes from *P. pastoris*. Given the ultra-high cell densities of *P. pastoris* cultures [13, 14], an approximately one order of magnitude higher yield may be expected by the present TAP purification from *P. pastoris*, as compared with that from *S. cerevisiae*.

The construct presented in this study contains a thrombin protease cleavage site (i.e., LVPRGS) between the C-terminus of each target protein and the THF tag, to facilitate tag removal after affinity purification (Fig. 1). Since we successfully obtained good single crystals of RNAP II using the ion-exchanged, THF-tagged protein fraction (Fig. 4), we did not test the cleavage of the THF tag by thrombin. The designed cleavage site, or any other protease recognition sequences, such as a TEV cleavage site (i.e., ENLYFQG), may be useful for the preparation of a tag-removed protein complex of interest.

In summary, we developed an efficient chromosome-tagging method in *P. pastoris* with a newly designed THF tag, and described rapid purification procedures for the large-scale preparation of endogenous multi-subunit protein complexes, through the examples of RNAP complexes I, II, and III. The protein complexes prepared by this method are highly homogeneous, and can be successfully utilized for crystallization, as demonstrated for RNAP II. Therefore, this method is generally applicable to the rapid large-scale preparation and high-resolution structural analyses of endogenous multi-subunit protein complexes from *P. pastoris*.

## Abbreviations

RNAP: RNA polymerase
TAP: Tandem affinity purification
TCEP: Tris(2-carboxyethyl)phosphine
SDS: Sodium dodecyl sulfate
PAGE: Polyacrylamide gel electrophoresis
CBB: Coomassie Brilliant Blue

## References

[1] Alberts B (1998). The cell as a collection of protein machines: preparing the next generation of molecular biologists. Cell.

[2] Mesa P, Deniaud A, Montoya G, Schaffitzel C (2013). Directly from the source: endogenous preparations of molecular machines. Curr Opin Struct Biol.

[3] Cramer P, Bushnell DA, Fu J, Gnatt AL, Maier-Davis B, Thompson NE, Burgess RR, Edwards AM, David PR, Kornberg RD (2000). Architecture of RNA polymerase II and implications for the transcription mechanism. Science.

[4] Groll M, Ditzel L, Löwe J, Stock D, Bochtler M, Bartunik HD, Huber R (1997). Structure of 20S proteasome from yeast at 2.4 Å resolution. Nature.

[5] Kerrigan JJ, Xie Q, Ames RS, Lu Q (2011). Production of protein complexes via co-expression. Protein Expr Purif.

[6] Imasaki T, Calero G, Cai G, Tsai KL, Yamada K, Cardelli F, Erdjument-Bromage H, Tempst P, Berger I, Kornberg GL, Asturias FJ, Kornberg RD, Takagi Y (2011). Architecture of the mediator head module. Nature.

[7] Rigaut G, Shevchenko A, Rutz B, Wilm M, Mann M, Séraphin B (1999). A generic protein purification method for protein complex characterization and proteome exploration. Nat Biotechnol.

[8] Puig O, Caspary F, Rigaut G, Rutz B, Bouveret E, Bragado-Nilsson E, Wilm M, Séraphin B (2001). The tandem affinity purification (TAP) method: a general procedure of protein complex purification. Methods.

[9] Bushnell DA, Kornberg RD (2003). Complete, 12-subunit RNA polymerase II at 4.1-Å resolution: implications for the initiation of transcription. Proc Natl Acad Sci USA.

[10] Fernández-Tornero C, Moreno-Morcillo M, Rashid UJ, Taylor NM, Ruiz FM, Gruene T, Legrand P, Steuerwald U, Müller CW (2013). Crystal structure of the 14-subunit RNA polymerase I. Nature.

[11] Engel C, Sainsbury S, Cheung AC, Kostrewa D, Cramer P (2013). RNA polymerase I structure and transcription regulation. Nature.

[12] Cregg JM, Cereghino JL, Shi J, Higgins DR (2000). Recombinant protein expression in *Pichia pastoris*. Mol Biotechnol.

[13] Cereghino GPL, Cereghino JL, Ilgen C, Cregg JM (2002). Production of recombinant proteins in fermenter cultures of the yeast *Pichia pastoris*. Curr Opin Biotechnol.

[14] Romanos MA, Clare JJ (1995) Culture of yeast for the production of heterologous proteins. Curr Protoc Protein Sci 2:5.8.1–5.8.17 10.1002/0471140864.ps0508s02 18429189

[15] Küberl A, Schneider J, Thallinger GG, Anderl I, Wibberg D, Hajek T, Jaenicke S, Brinkrolf K, Goesmann A, Szczepanowski R, Pühler A, Schwab H, Glieder A, Pichler H (2011). High-quality genome sequence of *Pichia pastoris* CBS7435. J Biotechnol.

[16] Leon S, Suriapranata I, Yan M, Rayapuram N, Patel A, Subramani S (2007). Characterization of protein-protein interactions: application to the understanding of peroxisome biogenesis. Methods Mol Biol.

[17] Gelbart ME, Rechsteiner TJ, Richmond TJ, Tsukiyama T (2001). Interactions of Isw2 chromatin remodeling complex with nucleosomal arrays: analyses using recombinant yeast histones and immobilized templates. Mol Cell Biol.

[18] Kushnirov VV (2000). Rapid and reliable protein extraction from yeast. Yeast.

[19] Ring GM, O’Connell MA, Keegan LP (2004). Purification and assay of recombinant ADAR proteins expressed in the yeast *Pichia pastoris* or in *Escherichia coli*. Methods Mol Biol.

[20] Kaneko A, Umeyama T, Hanaoka N, Monk BC, Uehara Y, Niimi M (2004). Tandem affinity purification of the *Candida albicans* septin protein complex. Yeast.

[21] Saade E, Mechold U, Kulyyassov A, Vertut D, Lipinski M, Ogryzko V (2009). Analysis of interaction partners of H4 histone by a new proteomics approach. Proteomics.

[22] Yang P, Sampson HM, Krause HM (2006). A modified tandem affinity purification strategy identifies cofactors of the *Drosophila* nuclear receptor dHNF4. Proteomics.

[23] Blethrow JD, Tang C, Deng C, Krutchinsky AN (2007). Modular mass spectrometric tool for analysis of composition and phosphorylation of protein complexes. PLoS One.

[24] Brummer MH, Richard P, Sundqvist L, Väänänen R, Keränen S (2001). The GDI1 genes from *Kluyveromyces lactis* and *Pichia pastoris*: cloning and functional expression in *Saccharomyces cerevisiae*. Yeast.

[25] Knop M, Siegers K, Pereira G, Zachariae W, Winsor B, Nasmyth K, Schiebel E (1999). Epitope tagging of yeast genes using a PCR-based strategy: more tags and improved practical routines. Yeast.

[26] Zhu B, Cai G, Hall EO, Freeman GJ (2007). In-fusion assembly: seamless engineering of multidomain fusion proteins, modular vectors, and mutations. Biotechniques.

[27] Wu S, Letchworth GJ (2004). High efficiency transformation by electroporation of *Pichia pastoris* pretreated with lithium acetate and dithiothreitol. Biotechniques.

[28] Kuhn CD, Geiger SR, Baumli S, Gartmann M, Gerber J, Jennebach S, Mielke T, Tschochner H, Beckmann R, Cramer P (2007). Functional architecture of RNA polymerase I. Cell.

[29] Kolodziej PA, Woychik N, Liao S-M, Young RA (1990). RNA polymerase II subunit composition, stoichiometry, and phosphorylation. Mol Cell Biol.

[30] Fernández-Tornero C, Böttcher B, Riva M, Carles C, Steuerwald U, Ruigrok RW, Sentenac A, Müller CW, Schoehn G (2007). Insights into transcription initiation and termination from the electron microscopy structure of yeast RNA polymerase III. Mol Cell.

